# Esophageal perforation secondary to malignant gastric outlet obstruction: a case report

**DOI:** 10.1186/s12957-019-1576-x

**Published:** 2019-02-19

**Authors:** Helen M. Johnson, Carlos J. Anciano, Shachar Laks

**Affiliations:** 1 Department of Surgery, Division of Surgical Oncology, East Carolina University Brody School of Medicine, Greenville, NC USA; 2 Department of Cardiovascular Sciences, Division of Thoracic Surgery, East Carolina University Brody School of Medicine, Greenville, NC USA

**Keywords:** Esophageal perforation, Boerhaave, Gastric outlet obstruction, Gastric cancer, Gastric adenocarcinoma, Esophageal stent, Endoscopic stent, Thoracoscopic drain, Transluminal endoscopy

## Abstract

**Background:**

Esophageal perforation is a rare presenting sign of gastric cancer. To date, only nine case reports of this phenomenon have been previously published.

**Case presentation:**

Esophageal perforation was diagnosed radiographically during workup for acute chest pain in a 67-year-old man. Emergent endoscopy confirmed esophageal perforation and biopsied a pre-pyloric mass confirmed to be adenocarcinoma. The perforation was managed with endoscopically placed transluminal pleural and mediastinal drains and esophageal stenting. The gastric outlet obstruction was temporized with a transpyloric stent. After the patient recovered from sepsis, distal gastrectomy was performed and he made a full recovery.

**Conclusions:**

Rarely, pre-pyloric gastric cancer can present with Boerhaave syndrome, spontaneous esophageal perforation associated with forceful vomiting. We present the tenth report in the literature of this phenomenon and the first to be initially treated with endoscopic stenting and transluminal thoracoscopic drainage. When endoscopic management is used to treat patients with Boerhaave syndrome, it may be beneficial to examine the entire stomach to evaluate for malignant etiology.

## Background

Gastric cancer is a major source of global burden of disease and mortality. Though it is the fifth most common malignancy worldwide, it is the third leading cause of cancer deaths [1]. The majority of cases are either locally advanced or metastatic at the time of diagnosis, with 5-year survival rates of 20–60% depending on the country [2]. Many patients are asymptomatic from the primary tumor or report nonspecific symptoms such as weight loss, abdominal pain, nausea, anorexia, and/or dysphagia [3]. Rarely, distal gastric cancer presents dramatically with esophageal perforation as the first sign of disease. To date, nine case reports of this unusual presentation have been published. Herein, we present the tenth reported case of esophageal perforation secondary to malignant gastric outlet obstruction and the first to be initially treated by endoscopic stenting and transluminal thoracoscopic drainage of the perforation and endoscopic stenting of the gastric outflow obstruction.

## Case presentation

**Fig. 1:**
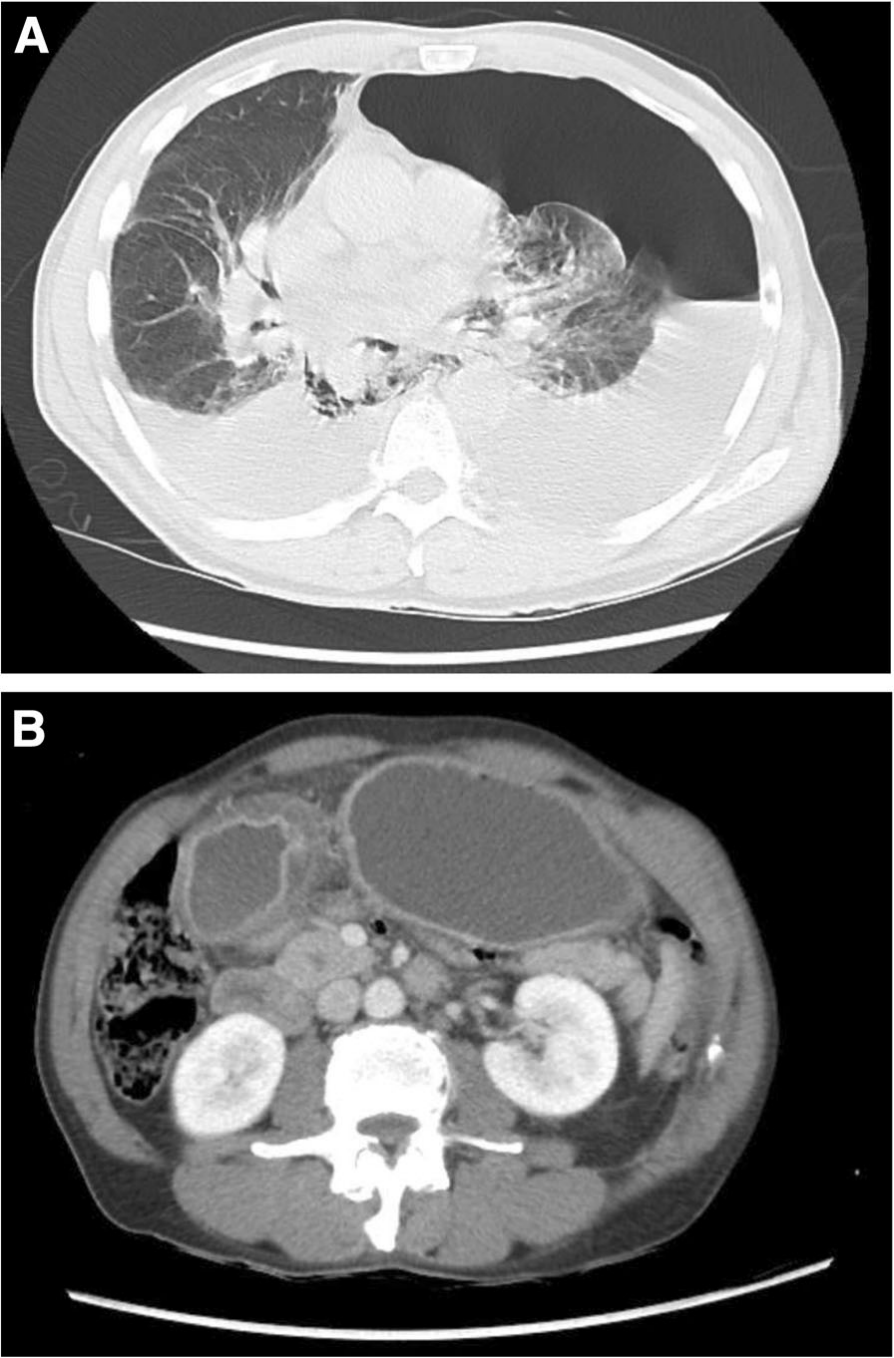
CT of the chest, abdomen, and pelvis with intravenous contrast. **a** Pneumomediastinum, left hydropneumothorax with rightward mediastinal shift, and right pleural effusion. **b** The distended, fluid-filled stomach with thickening of the pylorus consistent with gastric outlet obstruction

A 67-year-old man presented to the emergency room for acute chest pain, dyspnea, and chills. On further questioning, he reported 1 month of nausea, vomiting, and dysphagia and an unintentional 90-lb weight loss over the past year. Social history was notable for tobacco abuse of one pack per day and occasional alcohol use. Family history was significant for unspecified malignancy in both parents and a daughter, as well as gastric cancer in a brother and granddaughter. On exam, he was tachycardic (heart rate 110 beats/min), hypotensive (blood pressure 86/68 mmHg), and tachypneic (respiratory rate 40 breaths/min) and had diffuse left costal chest tenderness. Given his history of hypertension, cardiac workup was performed and was negative. Computed tomography (CT) of the chest was obtained and revealed an esophageal perforation and thickening of the gastric pylorus (Fig. 1), for which Thoracic Surgery was consulted. A left pleural 14-French (Fr) pigtail catheter was placed at the bedside to alleviate a large hydropneumothorax with tension component, and the patient was taken to the operating room emergently for endoscopic evaluation, wide drainage, and possible stenting.

**Fig. 2:**
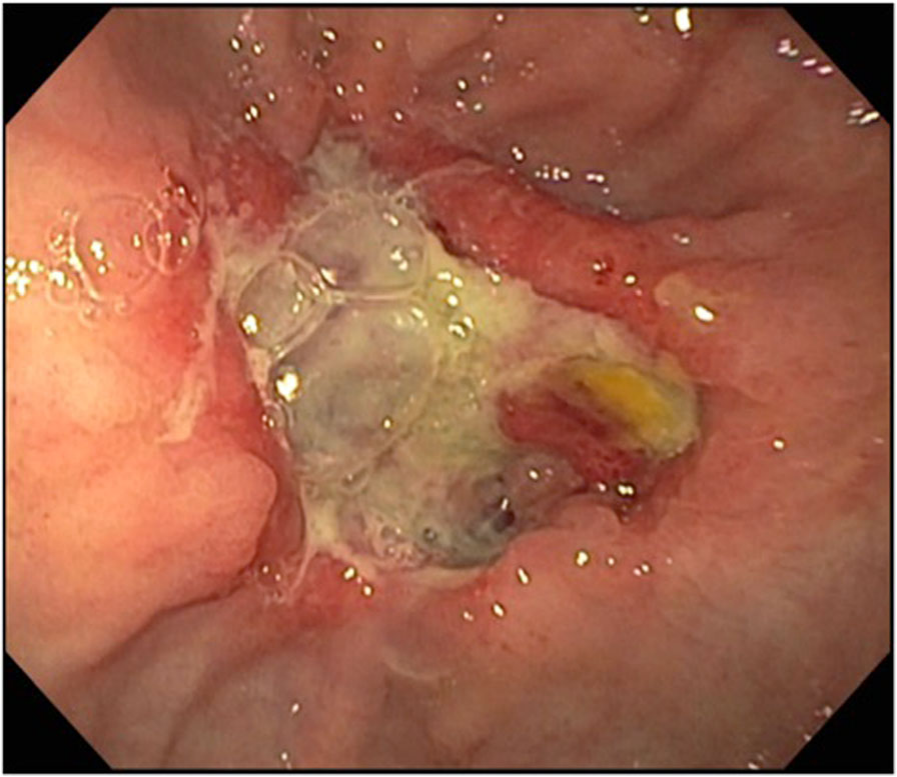
Pre-pyloric mass visualized on initial endoscopy

**Fig. 3:**
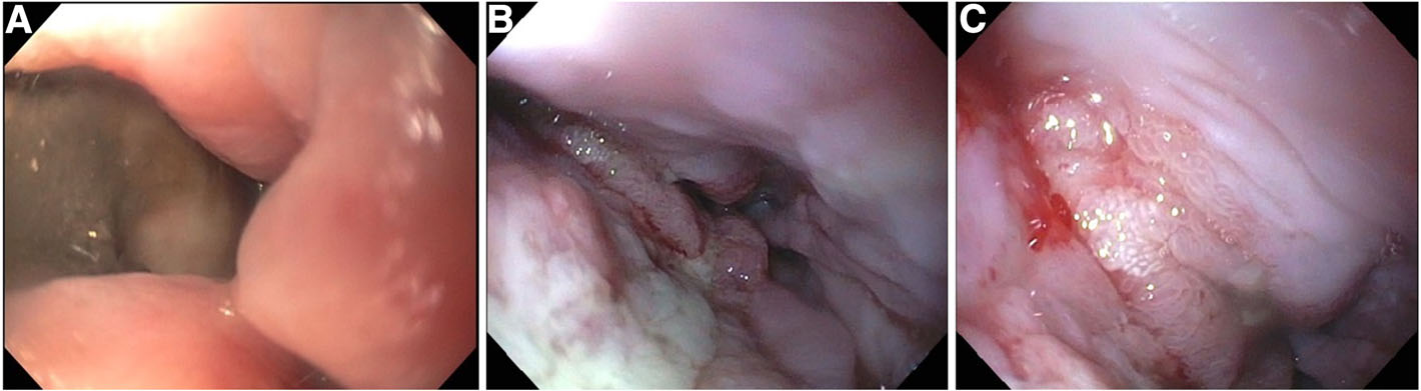
Serial endoscopic assessment of the esophageal perforation. **a** On initial endoscopy, a large distal esophageal perforation was visualized, with exposed mediastinum and pericardium. **b** After removal of the esophageal stent, the previous area of perforation was observed to be healing with healthy granulation tissue. **c** Subsequent follow-up endoscopy confirmed complete healing of the perforation

Esophagogastroduodenoscopy with fluoroscopy (Video 1 https://figshare.com/s/cbaef7705075bcb7a58f) showed a large perforation of the distal esophagus just proximal to the gastro-esophageal (GE) junction, a large fluid- and food-filled stomach, and a large ulcerative pre-pyloric mass (Fig. 2) which was biopsied. The esophageal perforation comprised approximately 30% of the circumference, was over 4 cm in length, and freely communicated with the posterior mediastinum and left pleural cavity (Fig. 3a). The endoscope was navigated across the perforation and passed alongside the pigtail catheter, using it to exteriorize the guidewire and endoscopic graspers. No 10 Jackson Pratt and 24-Fr Blake drains were then guided endoscopically across the chest wall into posterior mediastinum and sub-pulmonic pleural cavity directly adjacent to the luminal perforation to ensure wide, direct drainage. Bilateral percutaneous postero-apical pleural drainage tubes were then placed. The esophageal perforation was covered with a 23 × 120 mm fully covered stent (Alimaxx-ES, Merit Medical Systems, Utah). A nasogastric tube and 20-Fr percutaneous gastrostomy tube were placed to facilitate decompression in the setting of gastric outlet obstruction. Postoperatively, the patient was transferred to the intensive care unit where he was treated for septic shock with intravenous antimicrobial therapy and supportive care.

**Fig. 4:**
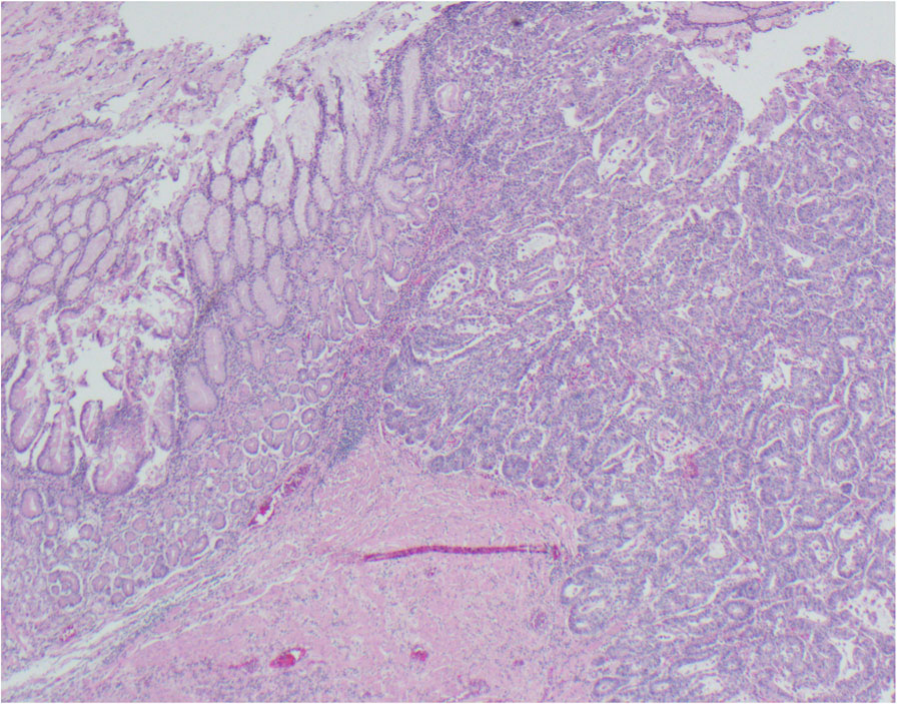
Pathologic confirmation of adenocarcinoma, intestinal type, in a background of gastric epithelium

The biopsy pathology and touch preparation cytology were discordant, so the patient was taken back to the operating room for repeat endoscopic biopsies when he was clinically stable. At this time, a temporary 23 × 100 mm covered stent was placed in a transpyloric position to alleviate the gastric outlet obstruction. Final pathology confirmed adenocarcinoma (Fig. 4).

Approximately 2 weeks later, the pyloric stent had started to migrate distally. In the absence of clinical and radiologic evidence of metastatic disease, surgical staging with possible concurrent oncologic resection was discussed with the patient and family, who elected to proceed. A distal gastrectomy with Billroth II reconstruction was performed, along with the removal of the temporizing transpyloric stent. Given the urgent nature of the procedure in a chronically ill patient still recovering from sepsis, extensive lymphadenectomy was not performed. Final pathology was pT2 pN1 (AJCC eighth edition), with a 1.8-cm moderately differentiated gastric adenocarcinoma with negative margins, and two of two lymph nodes evidencing metastatic disease including focal extra-nodal extension.

The patient required hospitalization for nearly 3 months for infection control, nutritional support, and physical rehabilitation. Four serial endoscopies were performed during this time for esophageal evaluation (Fig. 3b, c) and transluminal drain adjustments. The esophageal stent was noted to be in an appropriate position and was removed prior to discharge to a skilled nursing facility. After his functional status improved, the patient was referred to the Medical Oncology and Radiation Oncology for evaluation for adjuvant therapy for locally advanced disease. He is currently tolerating a regular diet and is fully ambulatory without support.

## Discussion and conclusions

This is the tenth reported case of esophageal perforation secondary to gastric outlet obstruction (GOO) from gastric cancer and the first to be temporized by esophageal and pyloric stenting with transluminal perforation management. Previously, Matsuhashi and colleagues [4] published the first English language case report of esophageal perforation secondary to malignant GOO. Their 2011 literature review identified five other similar cases. Our search yielded three additional cases [5–7]. Nine of the ten cases occurred in males, and the mean age was 63.2 years (range 47–73). All patients were treated with either distal or total gastrectomy, either concurrently with emergent surgical management of the esophageal perforation or after recovery from sepsis. The majority of patients were alive at the time of publication of their case report; however, follow-up times were quite variable (5 months to 6 years).

Significantly, the location of the tumor in all ten cases was pre-pyloric. The pathophysiology of the spontaneous esophageal perforations observed in these patients may be explained by malignant invasion of the pylorus inciting forceful vomiting akin to pyloric stenosis. Furthermore, it is likely that the stomach was unable to diffuse much of the force of retching as it was already maximally distended from months of accumulation of fluid and particulates secondary to poor gastric outflow.

Boerhaave syndrome, spontaneous esophageal rupture due to a sudden increase in intra-esophageal pressure often due to forceful vomiting, is a rare phenomenon with a mortality rate of about 40% [8]. Treatment options include surgical repair, operative drainage, endoscopic stenting, and non-operative management. Endoscopic management is gaining increasing popularity [9]. When this treatment method is chosen, it may be prudent to perform a complete esophagogastroduodenoscopy. Indeed, our case highlights the benefit of inspecting the gastrointestinal mucosa distal to the site of the perforation to evaluate for a malignant etiology.

## Abbreviations

CT: Computed tomography
Fr: French
GE: Gastro-esophageal
GOO: Gastric outlet obstruction
PET: Positron emission tomography
